# Structural and Functional Insight into Canarypox Virus CNP058 Mediated Regulation of Apoptosis

**DOI:** 10.3390/v9100305

**Published:** 2017-10-20

**Authors:** Mohd Ishtiaq Anasir, Amy A. Baxter, Ivan K. H. Poon, Mark D. Hulett, Marc Kvansakul

**Affiliations:** Department of Biochemistry and Genetics, La Trobe Institute for Molecular Science, La Trobe University, Melbourne, VIC 3086, Australia; 17818130@students.latrobe.edu.au (M.I.A.); A.Baxter@latrobe.edu.au (A.A.B.); i.poon@latrobe.edu.au (I.K.H.P.); m.hulett@latrobe.edu.au (M.D.H.)

**Keywords:** poxvirus, avipoxvirus, apoptosis, X-ray crystallography, isothermal titration calorimetry, Bcl-2

## Abstract

Programmed cell death or apoptosis is an important component of host defense systems against viral infection. The B-cell lymphoma 2 (Bcl-2) proteins family is the main arbiter of mitochondrially mediated apoptosis, and viruses have evolved sequence and structural mimics of Bcl-2 to subvert premature host cell apoptosis in response to viral infection. The sequencing of the canarypox virus genome identified a putative pro-survival Bcl-2 protein, CNP058. However, a role in apoptosis inhibition for CNP058 has not been identified to date. Here, we report that CNP058 is able to bind several host cell pro-death Bcl-2 proteins, including Bak and Bax, as well as several BH3 only-proteins including Bim, Bid, Bmf, Noxa, Puma, and Hrk with high to moderate affinities. We then defined the structural basis for CNP058 binding to pro-death Bcl-2 proteins by determining the crystal structure of CNP058 bound to Bim BH3. CNP058 adopts the conserved Bcl-2 like fold observed in cellular pro-survival Bcl-2 proteins, and utilizes the canonical ligand binding groove to bind Bim BH3. We then demonstrate that CNP058 is a potent inhibitor of ultraviolet (UV) induced apoptosis in a cell culture model. Our findings suggest that CNP058 is a potent inhibitor of apoptosis that is able to bind to BH3 domain peptides from a broad range of pro-death Bcl-2 proteins, and may play a key role in countering premature host apoptosis.

## 1. Introduction

Apoptosis is a form of programmed cell death that can be triggered via external (extrinsic pathway or receptor mediated) or internal stimuli (intrinsic pathway or mitochondria mediated). Apoptosis plays a major role in the removal of damaged, unwanted or infected cells, impacting processes ranging from cellular homeostasis to the immune response against viral infection [1]. In order to thwart host apoptosis as part of an antiviral response, viruses have evolved to encode arrays of apoptosis regulatory proteins [1]. For instance, many viruses express decoy receptors to neutralize the Tumor Necrosis Factor receptor superfamily, the effectors of extrinsic apoptosis pathway [1]. In addition, the host intrinsic apoptosis pathway can be downregulated by viruses through the expression of B-cell lymphoma 2 (Bcl-2) mimics (vBcl-2), which nullify the activity of host cell apoptosis inducing Bcl-2 family members [2,3].

The Bcl-2 family proteins are the gatekeepers of the intrinsic apoptosis pathway, with the family members being characterized by the presence of at least one of the four conserved Bcl-2 homology (BH) domains. Bcl-2 proteins can be divided into two major classes: pro-death and pro-survival [4]. The pro-death Bcl-2 proteins are further sub-divided into two groups; (1) pro-death Bcl-2, such as Bak and Bax that contain BH1-4 domains and (2) BH3-only proteins Bim, Bid, Puma, Bmf, Bik, Hrk, Noxa, and Bad that contain only the BH3 domain [4,5]. During cellular stress condition such as exposure to cytotoxic drugs, ultraviolet (UV) irradiation or viral infection, BH3-only proteins expression is up-regulated, resulting in the activation of Bak and Bax and/or neutralization of the pro-survival Bcl-2 proteins [6,7]. Upon their activation, Bak and Bax oligomerize to disrupt the outer mitochondrial membrane, facilitating the release of pro-apoptogenic factors such as cytochrome c, and ultimately triggering the caspase cascade and cell death [8]. The pro-survival Bcl-2 proteins include Bcl-2, Mcl-1, Bcl-x_L_, A1, Bcl-w, and Bcl-b, contain all four BH domains, and are responsible for antagonizing the pro-death Bcl-2 proteins and BH3-only proteins in order to maintain mitochondrial membrane integrity [9]. Structural studies revealed that Bcl-2 proteins interact with each other via a BH3 domain-hydrophobic groove dependent manner [10].

Many viruses have been found to encode vBcl-2 proteins including adenovirus E1B19K [11], African swine fever virus A179L [12], Epstein-Barr virus BHRF1 [13], and Kaposi’s Sarcoma herpes virus KSBcl-2 [14], which act either by directly binding and sequestering BH3 only proteins or by directly binding to Bak and Bax to prevent apoptosis initiation. Interestingly, poxviruses encode a series of highly diverse vBcl-2 proteins [14]. Sequence analysis showed that poxviral vBcl-2, such as Canarypox CNP058 and Fowlpox FPV039 harbor limited sequence identity with cellular Bcl-2, whilst others such as Vaccinia F1 and Myxoma M11L do not share any significant sequence identity [15]. Poxviral vBcl-2 proteins operate using several distinct mechanisms, for example, M11L inhibits apoptosis primarily by neutralizing Bak and Bax, whereas F1 targets Bim to inhibit apoptosis [16,17].

Canarypox CNP058 is a vBcl-2 protein discovered amongst certain avipoxviruses. In contrast to Fowlpox FPV039, which has been shown to adopt Bcl-2 fold [18] and is able to neutralize all BH3-only proteins, as well as Bak and Bax [18,19,20], little is known about CNP058. To understand the mechanism of apoptosis inhibition by CNP058, we measured the binding of CNP058 to peptides corresponding to BH3 domains of all pro-death Bcl-2 proteins, including Bak and Bax and all of the BH3-only proteins. We then determined the crystal structure of CNP058 bound to Bim BH3 domain, and demonstrate that CNP058 is able to potently inhibit UV light induced apoptosis in cell culture. Our findings demonstrate that CNP058 is a Bcl-2 protein that potently inhibits apoptosis, is able to interact with the BH3 domain of pro-death Bcl-2 proteins, and provide a platform to understand apoptosis inhibition by CNP058.

## 2. Materials and Methods

### 2.1. CNP058 Expression and Purification

Synthetic codon-optimized cDNA encoding for CNP058 lacking the C-terminal transmembrane domain (residues 1–143) (Genscript, Piscataway, NJ, USA) was cloned into the bacterial expression vector pGEX6P-1 (GE Healthcare, Chicago, IL, USA). The plasmid was transformed into *Escherichia coli* BL21 Star cells and grown in 2YT media supplemented with 1 mg/mL ampicillin. CNP058 was expressed using the auto-induction method for 24 h at 22 °C in a shaking incubator [21]. Bacterial cells were harvested by centrifugation at 3400× *g* (JLA 9.1000 rotor, Beckman Coulter Avanti J-E, Brea, CA, USA) for 15 min, resuspended in 100 mL lysis buffer (20 mM trisodium citrate pH 6.0, 200 mM NaCl), and lysed using a sonicator at 50 kHz for 4 cycles (15 s) with 30 s rest intervals in the presence of lysozyme (Sigma Aldrich, St. Louis, MO, USA) and DNAse I (deoxyribonuclease I from bovine pancreas, Sigma Aldrich). The resulting lysate was clarified by centrifugation at 31,000× *g* (JA 25.50 rotor, Beckman Coulter Avanti J-E) for 30 min. The supernatant was filtered with a 0.45 μM filter (Milipore, Burlington, MA, USA), loaded onto 5 mL of Glutathione Sepharose 4B resin (GE Healthcare) equilibrated with lysis buffer and subsequently washed with an additional 30 mL of lysis buffer. Human rhinovirus 3C protease (HRV3C protease) was added to the column and incubated overnight at 4 °C to liberate the target protein from the Glutathione-S-Transferase (GST) fusion tag. The cleaved target protein was eluted and concentrated to 5 mL prior to being subjected to size exclusion chromatography using a Superdex S75 16/600 column attached to an ÄKTAxpress system (GE Healthcare) equilibrated in 20 mM trisodium citrate pH 6.0, 200 mM NaCl. The protein eluted as a single peak and displayed higher than 95% purity based on sodium dodecyl sulfate-polyacrylamide gel electrophoresis (SDS-PAGE) analysis.

### 2.2. Measurement of Interactions with BH3 Peptides

Affinities of CNP058 for different synthetic BH3 peptides (Mimotopes, Mulgrace, VIC, Australia) were measured using a MicroCal iTC200 system (GE Healthcare) at 25 °C. The measurements were performed in 20 mM trisodium citrate pH 6.0, 200 mM NaCl at a final protein concentration of 30 μM. BH3 domain peptides at a concentration of 300 μM were titrated into the protein sample using 19 injections of 2 μL per injection. All of the assays were performed in triplicates. Protein concentrations were measured using a UV spectrophotometer (Thermo Scientific, Scoresby, VIC, Australia) at a wavelength of 280 nm. BH3 peptides concentrations were calculated from the dry weight of peptide. The BH3 peptide sequences used in this study were: (1) *Gallus gallus* (GG) GG_Bak (Uniprot Id: Q5F404): 72-LGSTGSQVGRRLAIIGDDINKRYDAE-97; (2) *Homo sapiens* (HS) HS_Bax (Uniprot Id: Q07812): 50-VPQDASTKKLSECLKRI GDELDSNMELQ-77; (3) GG_Bid (Uniprot Id: Q8JGM8): 77-PEVNEAIVRTIAAQLA EIGDQLDKQIKAKVVNDL-110; (4) GG_Bmf (Uniprot Id: A9XRG9): 135-EARTEVQIARKLQCIADQFHRLHIQR-160; (5) GG_Bok (Uniprot Id: Q9I8I2): 67-VSAILLRLGDELEYIRPNVYRNIARQ-92; (6) GG_Noxa (RefSeq Id NP_001289026.1: ERDAVAECALELRRIGDKADLQQKVL; (7) GG_Bik (Uniprot Id: E9JEC5): 43-ISSAIQVGHQLALIGDEFNRAYSRK-67; (8) HS_Bim (Uniprot Id: O43521-3): 51-DMRPEIWIAQELRRIGDEFNAYYARR-76; (9) HS_Puma (Uniprot Id: Q9BXH1-1): 130-EEQWAREIGAQLRRMADDLNAQ-YERR-155; (10) HS_Hrk (Uniprot Id: O00198-1): 26-RSSAAQLTA ARLKAIGDE-LHQRTMWR-51; (11) HS_Bad (Uniprot Id: Q92934-1): 103-NLWAAQRYGRELRRMSDEFVDSFKKG-128.

### 2.3. CNP058 Complex Crystallization and Data Collection

A complex of CNP058 with the Bim BH3 domain was prepared as previously described [22]. Briefly, CNP058-Bim BH3 complex was reconstituted by adding HS_Bim BH3 domain at a 1:1.25 molar ratio to CNP058. The reconstituted complex was used for crystallization trials using 96-well sitting drop trays (Swissci, Neuheim, Switzerland) with the vapour diffusion method at 20 °C. A total of 0.15 μL CNP058-Bim BH3 domain peptide complex was mixed with 0.15 μL of various crystallization conditions using a Gryphon nanodispenser robot (Art Robbins, Sunnyvale, CA, USA). Commercially available screening kits (Crystal Screen, PACT suite, JCSG-plus Screen and PEG/Ion Screen) were used as prepared by C3 for the initial crystallization screening, with hit optimization performed using 24-well hanging drop plates (EasyXtal DG-Tool, Qiagen, Hilden, Germany) of 1 + 1 μL protein:reservoir condition.

Crystals of CNP058 in complex with the Bim BH3 domain were obtained at 10 mg/mL in 0.2 M calcium chloride dihydrate, 0.1 M MES pH 6.0, 20% (*w*/*v)* PEG6000. This condition produced thick needle crystals of CNP058-Bim BH3 domain complex belonging to the C2 space group in the monoclinic crystal system. The final crystal contained one molecule of CNP058 bound to one molecule of Bim BH3 in the asymmetric unit and had a solvent content of 40.1%. The crystals were cryoprotected using 20% (*v*/*v*) ethylene glycol, and was flash cooled at 100 K using liquid nitrogen. All diffraction data were collected on the MX2 beamline at the Australian Synchrotron using an Eiger detector with an oscillation range of 0.1° per frame using a wavelength of 0.9537 Å. Diffraction data were integrated using XDS [23] and scaled using AIMLESS [24,25]. The structure of the CNP058-Bim BH3 domain complex was solved using molecular replacement with Phaser [26], with the structure of FPV039 (PDB: 5TZP) as a search model [18]. The final TFZ and LLG values were 8.9 and 102, respectively. The solution produced by Phaser was manually rebuilt over multiple cycles using Coot [27] and refined using PHENIX [28]. Data collection and refinement statistics details are summarized in Table 1. Coordinate files have been deposited in the Protein Data Bank under the accession code 5WOS. All of the images were generated using the PyMOL Molecular Graphics System, Version 1.8 Schrödinger, LLC, New York, NY, USA). All of the software was accessed using the SBGrid suite [29,30]. All of the raw diffraction images were deposited on the SBGrid Data Bank [30] using accession numbers 5WOS.

### 2.4. Cell Culture

Human epithelial cervical cancer (HeLa) cells were cultured in RPMI-1640 medium (Invitrogen, Carlsbad, CA, USA). All culture media were supplemented with 10% fetal bovine serum, 100 U/mL penicillin, and 100 μg/mL streptomycin (Invitrogen). Cell lines were cultured at 37 °C in a humidified atmosphere containing 5% CO_2_ and detached from the flask with 0.25% trypsin and 0.5 μM EDTA (Invitrogen).

### 2.5. Transfection of HeLa Cells with GFP Constructs and Induction of Apoptosis

Full length CNP058 (residues 1–175) was cloned into the mammalian expression vector pcDNA3.1+N-eGFP (Genscript), vaccinia virus (WR) F1 in pEGFP-C3 was provided by Michele Barry (University of Alberta, Edmonton, AB, Canada) [31]. HeLa cells were grown to 60% confluency and transfected with plasmid constructs for green fluorescent protein (GFP)-tagged F1, GFP-tagged CNP058 or cytosolic GFP using X-tremeGENE 9 DNA Transfection Reagent (Roche, Basel, Switzerland) as per manufacturer’s instructions. 24 h post transfection, cell supernatants were replaced with fresh growth media and cells were subjected to ultraviolet (UV) irradiation using a Stratalinker UV crosslinker (Stratagene, La Jolla, CA, USA), followed by 6 h incubation at 37 °C in a humidified atmosphere containing 5% CO_2_.

### 2.6. Flow Cytometry

A two stain flow cytometry based apoptotic cell death assay, described previously in [32], was performed to analyze the ability of virus-encoded apoptosis inhibitors to inhibit apoptosis in HeLa cells subjected to UV irradiation [32]. Briefly, following apoptosis induction, trypsinized cells were stained with PE-conjugated annexin V stain (AV-PE) (BD Biosciences, San Jose, CA, USA) and TO-PRO-3 nucleic acid stain (Life Technologies, Carlsbad, CA, USA) as per the manufacturer’s instructions and kept on ice until analysis by flow cytometry using the BD FACSCanto II Flow Cytometer and BD FACSDiva software v6.1.1 (BD Biosciences, St. Jose, CA, USA). Samples were subsequently analyzed with Flowjo software v8.8.6 (Tree Star, Ashland, OR, USA). A minimum of 9000 GFP-positive cell events were recorded per sample, with GFP-negative cells excluded from analysis and AV-PE/TO-PRO-3 staining used to gate apoptotic from non-apoptotic cell populations [32].

## 3. Results

**Figure 1:**
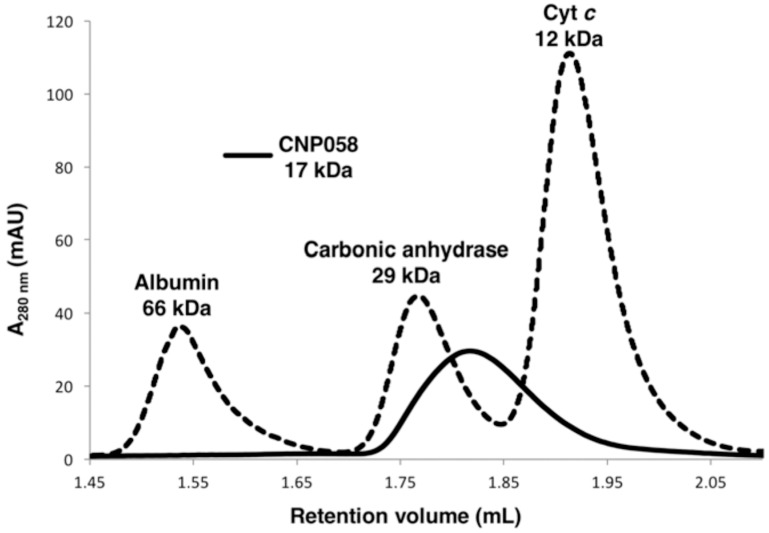
CNP058 is a monomer in solution. Size exclusion chromatography of CNP058 using a Superdex 200 Increase 3.2/300 column. The elution volume of the peak of interest (CNP058) is 1.77 mL (solid line). The molecular weight standards shown are albumin (66 kDa), carbonic anhydrase (29 kDa) and cytochrome *c* (Cyt *c*) (12 kDa) (all source from Sigma Aldrich), shown as dotted lines and labeled on top of the respective peaks. AU: absorbance units.

To examine if CNP058 is able to interact with pro-death Bcl-2, truncated recombinant CNP058 encompassing the first 143 amino acids was expressed in *E. coli* and purified using a two step purification method of affinity chromatography followed by size exclusion chromatography. Since a number of vBcl-2 proteins have been shown to be dimers in solution, we examined size exclusion chromatograms to define the oligomeric state of CNP058 (Figure 1). Our results show that CNP058 is a monomer in solution.

**Figure 2:**
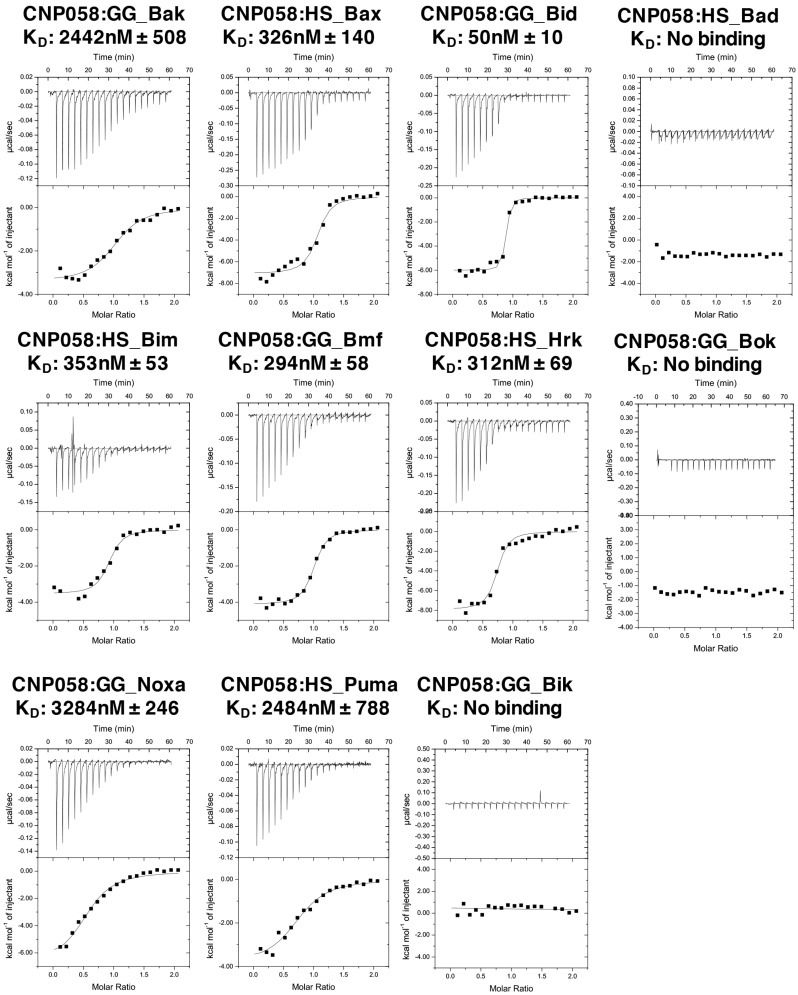
CNP058 interacts with Bak and Bax as well as all BH3-only proteins except Bad and Bik. Raw heats measured by isothermal titration calorimetry (ITC) for CNP058 interactions with Bcl-2 homology domain 3 (BH3) domain peptides of pro-death B-cell lymphoma 2 (Bcl-2) proteins from *Homo sapiens* (HS) and *Gallus gallus* (GG).

We then examined binding of CNP058 to peptides comprising the BH3 domain of all pro-death Bcl-2 proteins using isothermal titration calorimetry (ITC) (Figure 2). ITC showed that CNP058 engages BH3 domains of Bid with high affinity (50 nM), whereas Bax, Bim, Hrk, Bmf interacted with 7–9-fold weaker affinity, and Bak, Puma and Noxa only displayed affinity in the micromolar region (Figure 2). In contrast, Bik, Bad, and Bok did not show any detectable affinity for CNP058.

**Figure 3:**
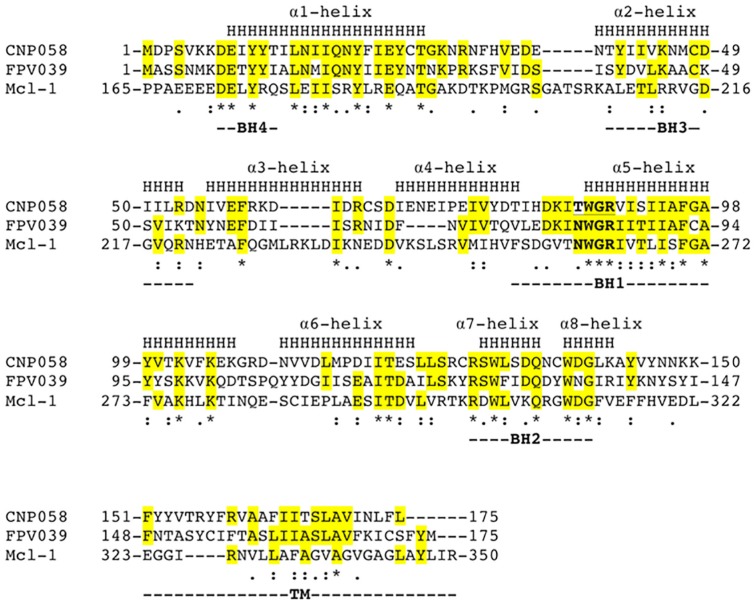
Structure based sequence alignment of CNP058 with FPV039 and cellular Bcl-2 protein Mcl-1. The α-helical secondary structure elements indicated (denoted as H and labeled as Helix 1–8) are based on the crystal structure of CNP058. Bcl-2 homology domains (BH domains) 1–4 are marked underneath the aligned sequences. Conserved residues are highlighted in yellow. “*” denotes conserved residues for all three proteins, “:” denotes highly similar residues between the three proteins, and “.” denotes weakly similar residues between the three proteins. The NWGR or TWGR motifs are marked in bold. Uniprot accession codes: CNP058 = Q6VZT9, FPV039 = Q9J5G4 and Mcl-1 = Q07820; PDB accession codes: FPV039 = 5TZP and Mcl-1 = 2NL9.

**Figure 4:**
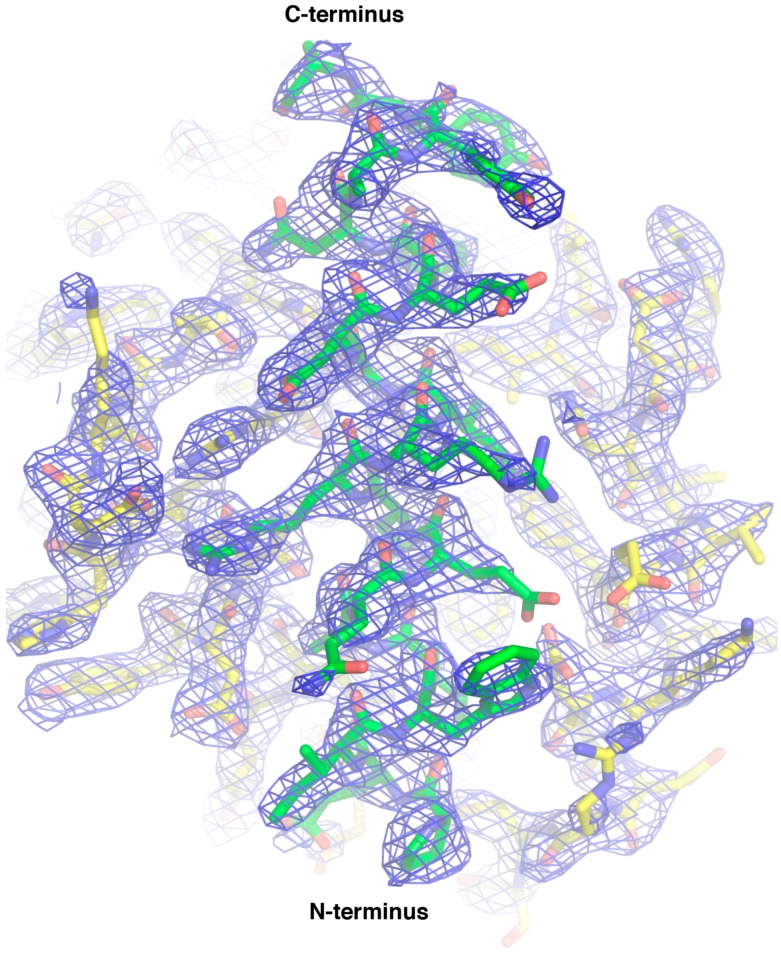
2Fo-Fc electron density maps of CNP058:Bim BH3 domain complex. Electron density map encompassing the hydrophobic binding groove of CNP058 in complex with Bim BH3. CNP058 is shown as yellow sticks, whereas Bim BH3 is shown as green sticks. The electron density map is shown as a blue mesh contoured at 1 δ.

**Figure 5:**
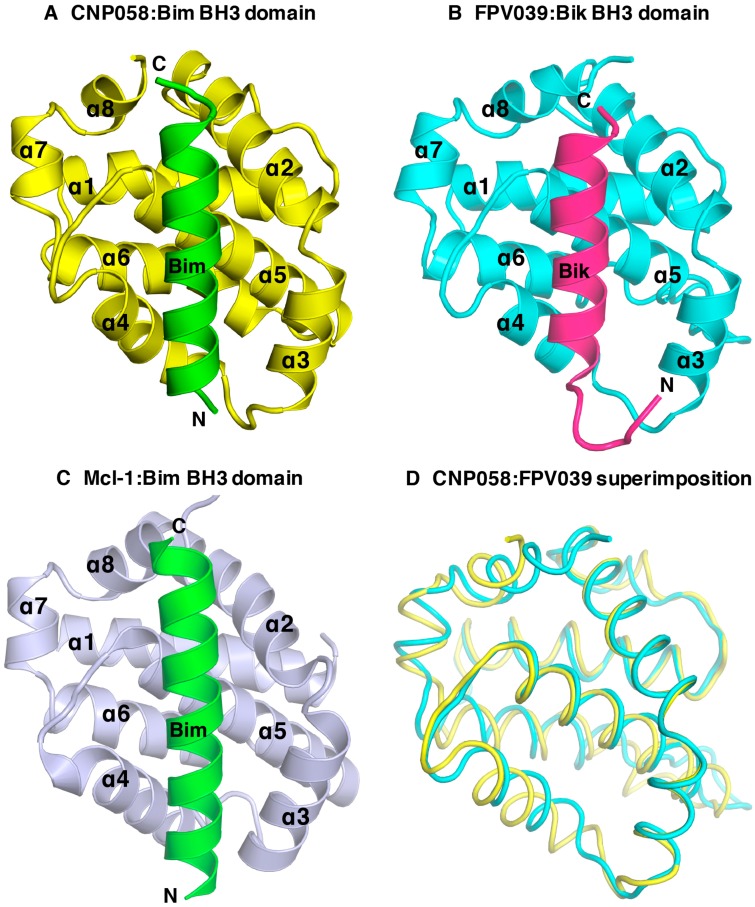
CNP058 interacts with pro-death Bcl-2 proteins in a BH3-domain hydrophobic groove dependent manner. (**A**) Cartoon representation of CNP058:Bim BH3 domain complex, (**B**) FPV039:Bik BH3 domain complex and (**C**) Mcl-1:Bim BH3 domain complex. The view is into the conserved hydrophobic ligand binding groove formed by α-helices 2–5. PDB ID: FPV039:Bik = 5TZP, Mcl-1:Bim = 2NL9. (**D**) CNP058:Bim complex (yellow) was superimposed onto FPV039:Bik complex (cyan). Bim and Bmf were removed from respective complex structures for clarity.

We then sought to understand the structural basis of CNP058 interaction with the BH3 domain of pro-death Bcl-2 by determining the structure of CNP058 in complex with the Bim BH3 domain. The CNP058-Bim BH3 domain complex structure revealed that CNP058 adopts the conserved Bcl-2-like fold consisting of eight α-helices and possessing the conserved hydrophobic groove found in other Bcl-2 family members that is utilized to accommodate Bim BH3 domain (Figure 3, Figure 4 and Figure 5A, Table 1). The closest cellular Bcl-2 structural homolog as identified by a DALI search is Mcl-1 (root mean square deviation (r.m.s.d)) of 1.0 Å over 94 Cα atoms, Figure 5C) and the closest viral Bcl-2 homolog is FPV039 (r.m.s.d. of 1.2 Å over 114 Cα atoms, Figure 3 and Figure 5B,D).

**Figure 6:**
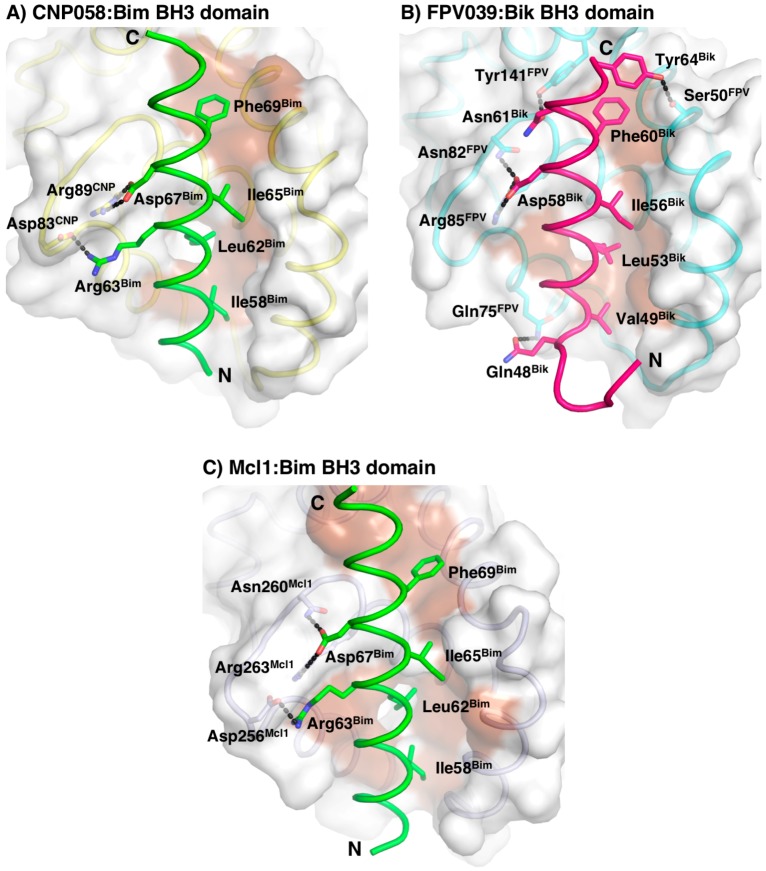
CNP058:Bim BH3 complex interface. Surface representation of (**A**) CNP058:Bim BH3 domain complex, (**B**) FPV039:Bik BH3 domain complex, and (**C**) Mcl-1:Bim BH3 domain complex. The view is into the conserved hydrophobic ligand binding groove formed by α-helices 2–5. The surfaces are shown in grey. Residues involved in interactions are shown as sticks and labeled. Hydrogen bonds and ionic interactions between CNP058 and Bim are denoted as black dotted lines. PDB ID: FPV039:Bik = 5TZP, Mcl-1:Bim = 2NL9.

The Bim BH3 domain is bound in the canonical hydrophobic ligand binding groove formed by α-helices 2–5 (Figure 6). Detailed inspection of the CNP058-Bim BH3 domain complex interface (Figure 6A) showed that Bim residues Ile58, Leu62, Ile65 and Phe69 extend into four hydrophobic pockets on the CNP058 binding groove. In addition, two salt bridges are found at the interface: Asp83^CNP058^-Arg63^Bim^ and the highly conserved Arg89^CNP058^-Asp67^Bim^, which is a hallmark of pro-survival Bcl-2:BH3 domain interactions. Notably, the highly conserved **NWGR** motif situated at the beginning of α5-helix of all Bcl-2 proteins is replaced with a **TWGR** motif in CNP058, however the Thr in the TWGR motif is not involved in any direct interactions with Bim.

**Figure 7:**
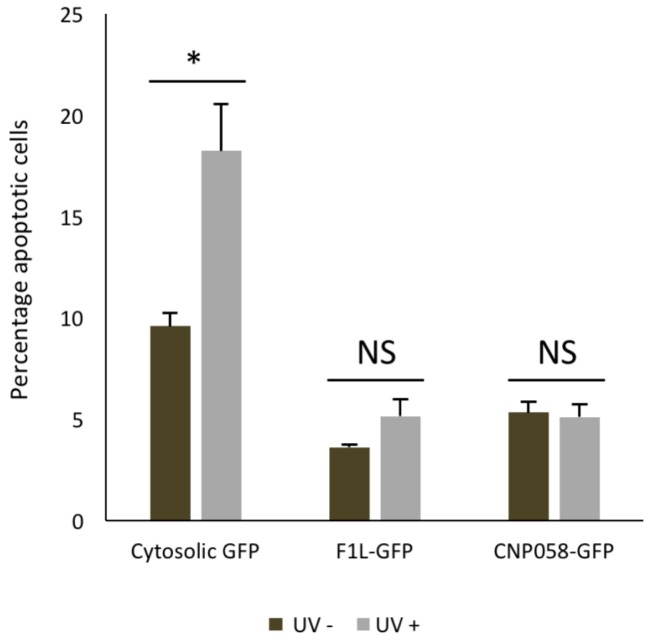
CNP058 is a potent inhibitor of UV induced apoptosis. The ability of HeLa cells expressing green fluorescent protein (GFP) tagged virus encoded apoptosis inhibitors CNP058 or F1 or cytosolic GFP to undergo apoptosis was determined 6 h after UV irradiation via flow cytometry. The percentage of apoptotic cells in total GFP-positive cell population was determined by AV-PE/TO-PRO-3 staining. Data are representative of three independent experiments, error bars represent the standard error of the mean (SEM), *n* = 3. Unpaired Student’s two-tailed *t*-test was performed in Excel, * *p* < 0.05, NS: non-significant.

To determine the functional relevance of CNP058’s ability to engage peptides from pro-apoptotic Bcl-2 proteins, we then examined the capacity of CNP058 to inhibit apoptosis in HeLa cells triggered by UV irradiation (Figure 7). We observed that CNP058 acts as a potent inhibitor of UV induced apoptosis in HeLa cells when compared to the prototypical poxviral apoptosis inhibitor F1 from Vaccinia virus.

## 4. Discussion

Premature host cell apoptosis is a crucial mechanism as part of an innate response against viral infection to limit spread of viral infections. Consequently, many viruses including adenoviruses [11,33], herpesviruses [14] and poxviruses [18,34] evolved to encode vBcl-2 proteins that are important to subvert Bcl-2-mediated host cell apoptosis. Functional studies revealed that poxviruses encode a wide range of vBcl-2 proteins harboring diverse modes of action to interfere with apoptosis signaling [3,16,20,34]. For example, vaccinia virus F1 and deerpox virus DPV022 possess a very restricted binding profile where both only engage Bim with high affinity [34,35] (Table 2). In contrast, fowlpox virus FPV039, ORF virus ORFV125, and sheeppox virus SPPV14 target a wider range of cellular Bcl-2 proteins: FPV039 engages all BH3-only proteins and Bak and Bax [18,20], ORFV125 binds Bim, Bik, Hrk, Noxa, Puma, and Bax [36], whereas SPPV14 binds Bim, Bid, Bmf, Hrk, Puma, Bak and Bax [37].

CNP058 was identified as a hypothetical vBcl-2 in the canarypox genome that shares limited sequence identity with Mcl-1 [49] (Figure 3). Other vBcl-2 proteins identified in avipox genomes includes fowlpox FPV039, pigeonpox vBcl-2, penguinpox vBcl-2 and turkeypox vBcl-2 [18]. Phylogenetic analysis based on the sequences of the core 4b protein, DNA polymerase as well as vBcl-2 revealed that avipoxviruses are divided into two clades: fowlpox virus-like and canarypox virus-like [18,50,51]. Unlike FPV039, which is the prototypical vBcl-2 member of the fowlpox virus-like clade and has previously been shown to adopt Bcl-2-like fold and inhibits apoptosis by targeting all BH3-only proteins as well as Bak and Bax [18,19,20], not much is known about apoptosis regulation by CNP058, the prototypical vBcl-2 from the canarypox virus-like clade. To address this, we examined the ability of CNP058 to bind BH3 domains of all pro-death Bcl-2 proteins (Figure 2). Interestingly, CNP058 only displayed a high binding affinity towards Bid (50 nM), whilst Bim, Hrk, Bax, and Bmf were bound with moderate affinities of 353 nM, 312 nM, 326 nM and 294 nM, respectively. Furthermore, CNP058 bound the BH3 domains of Bak, Noxa, and Puma with low affinities of 2442 nM, 3284 nM, and 2484 nM, respectively. The binding profile for pro-apoptotic Bcl-2 proteins of CNP058 is in marked contrast to the one previously reported for FPV039, which demonstrated that it binds to both Noxa and Bad, an unusual feature not seen in cellular pro-survival Bcl-2 proteins, which only bind either Bad or Noxa [18]. Interestingly, despite sharing a high sequence similarity with FPV039 (38% identity, 58% similarity, Figure 3), CNP058 only displays binding to Noxa and not Bad, thus more resembling the binding profile of cellular pro-survival Bcl-2 proteins Mcl-1 and A1 [18]. The inability of CNP058 to interact with Bad BH3 domain suggests that Bad BH3 mimetics ABT-737 and ABT-263 would not be useful compounds to inhibit CNP058 in birds infected by canarypox virus, as these drugs are specific for pro-survival Bcl-2 proteins that interact with Bad BH3 [52]. Another interesting feature of the CNP058 pro-death Bcl-2 binding profile is the fact that it interacts with Noxa. To date only FPV039, A179L and ORFV125 have been identified as vBcl-2 proteins that interact with Noxa [18,36,39]. ORFV125 has been shown to interact with Noxa through immunoprecipitation experiment, however the affinity of the interaction has not yet been established, whereas FPV039 binds Noxa with tightly with an affinity of 28 nM, whilst the binding of A179L is of more modest affinity (1575 nM), as established using ITC [18,36,39]. Noxa has been shown to be upregulated in the presence of viral infection, double stranded RNA, and interferon, suggesting Noxa as a viral sensor to activate host intrinsic apoptosis [6,53]. The interaction of FPV039 and CNP058 with Noxa suggest that avipoxviruses may counter host intrinsic apoptosis triggered by Noxa during viral infection.

Despite being closely related to FPV039, a comparison of the binding profiles showed that CNP058 displays a distinct interaction profile with pro-death Bcl-2 proteins. CNP058 is similar to other vBcl-2, which only bind to a select subset of pro-death Bcl-2 proteins, whereas FPV039 and A179L are highly promiscuous and are able to engage all major pro-apoptotic Bcl-2 proteins [18,39]. In addition, the binding affinities of CNP058 towards pro-death Bcl-2 BH3 domains are significantly lower when compared to FPV039, with only the interaction of CNP058 with Bid in the low nanomolar range. In contrast, nearly all of the cellular pro-death BH3 domains interacted tightly with FPV039 with a high nanomolar affinity [18]. However, despite displaying substantially weaker affinities for peptides from all of the identified endogenous pro-apoptotic Bcl-2 members, we demonstrate that CNP058 is a potent inhibitor of UV irradiation induced apoptosis in transfected HeLa cells, and appears to be an equally potent inhibitor of apoptosis when compared to the prototypical poxviral pro-survival Bcl-2 protein F1 from vaccinia virus (Figure 7) [17,31,54,55,56]. However, when considering that our apoptosis assays were performed using cells transfected with CNP058 in the absence of infectious virus, the functional relevance for the comparable potency of apoptosis inhibition efficiency by F1 and CNP058 remain to be established.

**Figure 8:**
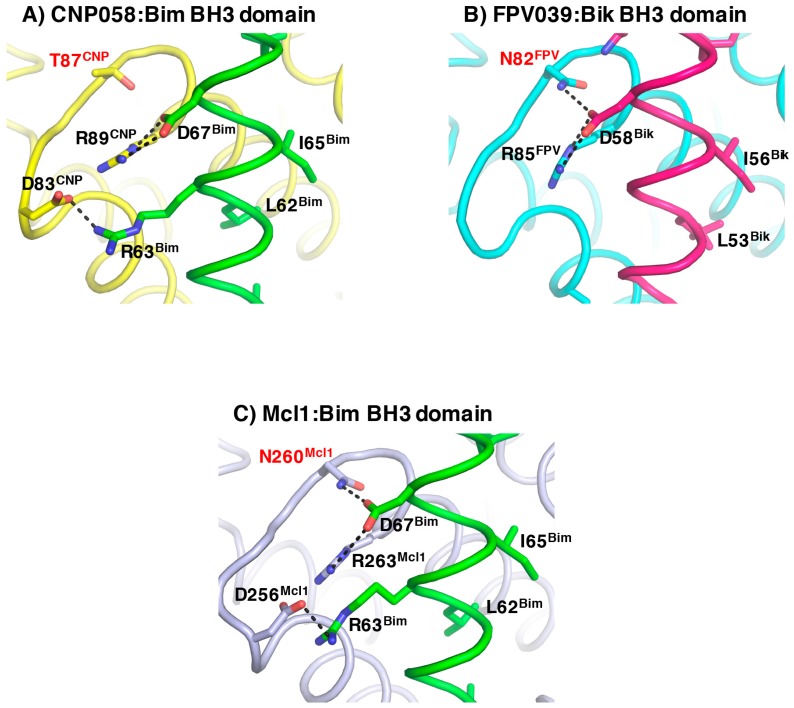
The role of the conserve NWGR motif. Detailed cartoon representation of the NWGR or equivalent motif in (**A**) CNP058:Bim BH3 domain complex, (**B**) FPV039:Bik BH3 domain complex and (**C**) Mcl-1:Bim BH3 domain complex. The view is into the conserved hydrophobic ligand binding groove formed by α-helices 2–5. Residues involved in interactions are shown as sticks and labelled. Hydrogen bonds and ionic interactions between pro-survival proteins and pro-apoptotic ligands are denoted as black dotted lines. The Asn residues in the NWGR motif in FPV039 and Mcl-1 as well as the Thr in the TWGR motif are labelled in red. PDB ID: FPV039:Bik = 5TZP, Mcl-1:Bim = 2NL9.

At the structural level, CNP058 possesses longer α4, α5, and α6 helices in comparison to FPV039 (Figure 5D) [18]. These topological differences may contribute to the distinct binding profiles of the two proteins as α4 and α5 helices form core segments of the hydrophobic binding groove. Furthermore, the substitution of an Asn in the highly conserved **NWGR** for Thr may cause the difference in the binding profile between the two avipoxvirus vBcl-2s, since the threonine in **TWGR** motif of CNP058 is unable to form hydrogen bonds with bound BH3 domains, whereas the corresponding Asn in FPV039 is involved in hydrogen bonds in complexes with Bik and Bmf [18] (Figure 8). This suggests that the Asn in the **NWGR** motif is an important determinant for the interactions at the very least for avipox encoded pro-survival Bcl-2 proteins with the pro-death BH3 domains, a notion that is supported by similar findings for human Bcl-b [57], which binds only Bim and Bax amongst the cellular pro-apoptotic Bcl-2 proteins, in both cases with low half maximal inhibitory concentration (IC_50_) values of 6.6 and 113 μM. Furthermore, the mouse Bcl-2 protein Boo features a SWSQ instead of the typical NWGR motif, and is unable to engage any pro-apoptotic Bcl-2 proteins [58], further underscoring the impact that substitutions in the NWGR motif have on pro-survival Bcl-2 proteins and their ability to bind to their pro-apoptotic cellular counterparts.

The overall structure of CNP058 is similar to other cellular Bcl-2 proteins, with an extended loop region connecting the α1 and α2 helices, a feature only seen in the two other vBcl-2 proteins, Bhrf1 and FPV039 [18,40]. However, CNP058 features a short loop region connecting helices α5–6, which is in marked contrast to the other avipoxviral Bcl-2 protein FPV039. Whereas, the α5–α6 loop in FPV039 was extended and comprised of 16 residues, the equivalent section in CNP058 features only a 3 residue loop, but significantly longer α5 and α6 helices. The impact of this difference on the ability of CNP058 to engage pro-apoptotic Bcl-2 proteins is not immediately obvious since this region is somewhat distant from the canonical ligand binding groove, and is thus not likely to be a significant determinant of pro-apoptotic Bcl-2 binding profiles.

**Figure 9:**
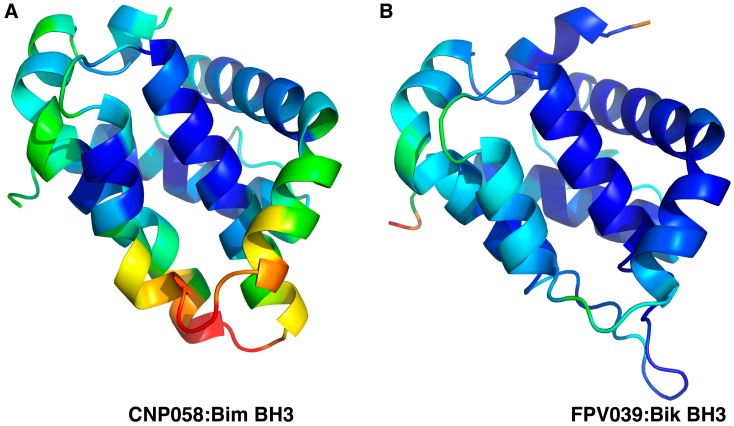
Cartoon representation of B-factor distribution of Cα atoms in (**A**) CNP058:Bim BH3 domain complex, (**B**) FPV039:Bik BH3 domain complex The view is into the conserved hydrophobic ligand binding groove formed by α-helices 2–5. BH3 domain ligands were removed for clarity. Colour are based on residues with low B-factor (blue) to high B-factor (red). PDB ID: FPV039:Bik = 5TZP.

A comparison of the mode of ligand binding between the CNP058:Bim and FPV039:Bik complex reveals that binding of Bim to CNP058 buries a total of 1700 Å^2^ of solvent accessible surface and an associated ∆*G* of interface formation and dissociation of −11.1 and 4.5 kcal/mol, respectively, whereas binding of Bik to FPV039 buries a total of 1450 Å^2^ and an associated ∆*G* of interface formation and dissociation of −8.9 and 2.9 kcal/mol, respectively, suggesting that ligand binding to CNP058 is driven more by hydrophilic interactions as compared to FPV039. Furthermore, we examined the B-factor distribution along the key α3 and α4 helices that form the walls of the ligand binding groove in both CNP058 and FPV039 (Figure 9). Interestingly, in CNP058, significant B-factor differences are observed for the entire α3 helix and the lower part of helix α4 compared to the central helix α5, suggesting that both α3 and α4 are display considerably higher flexibility. In contrast, in the FPV039-Bik complex, only helix 4 displays higher B-factors when compared to the central helix α5, whereas helix α3 does not display significantly higher thermal motion.

Amongst the poxviruses, a number of sequence, structural, and functional homologs of Bcl-2 have been identified to date. These include proteins that displayed little or no over sequence identity with cellular Bcl-2 proteins such as myxoma virus M11L, deerpox virus DPV022, sheeppox virus SPPV14, variola virus F1, and vaccinia virus F1, N1, A49, A52, B14, and K7 [17,34,35,38,59,60,61,62]. These poxviral vBcl-2 proteins can be divided into three groups: (1) monomeric pro-survival vBcl-2 such as M11L, (2) dimeric vBcl-2 such as F1 and DPV022, and (3) A49, A52, B14, and K7, which all were identified as vBcl-2 proteins after their structures were determined experimentally, that do not interact with pro-death Bcl-2 and carry out other immunomodulatory functions such as nuclear factor kappa-light-chain-enhancer of activated B cells (NF-κB) pathway and interferon regulatory transcription factor 3 (IRF3) inhibitors [16,34,35,60,61,62]. Vaccinia virus N1 is unusual since it is dimeric, and functions as a dual inhibitory protein that modulates both intrinsic apoptosis and NF-κB signaling. Based on biochemical and structural studies, CNP058 and FPV039 are distinct from other poxvirus encoded vBcl-2 proteins since they both display readily identifiable sequence identity with cellular Bcl-2 proteins due to their obvious BH1 and BH2 motifs. Furthermore, at the structural level, they both closely resemble the cellular pro-survival protein Mcl-1. This suggests that FPV039 and CNP058 may be the closest homologs of cellular Bcl-2 proteins within the poxviruses.

In summary, our findings reveal that CNP058 adopts a Bcl-2-like fold similar to cellular pro-survival protein Mcl-1 and its avipoxvirus vBcl-2 counterpart FPV039. We showed that CNP058 binds to BH3 domains of pro-death proteins including Bak and Bax and most of the BH3-only proteins, and is able to protect HeLa cells against UV induced apoptosis. These findings establish CNP058 as a viral Bcl-2 protein with a broad pro-apoptotic Bcl-2 binding profile that is a potent inhibitor of apoptosis.

## Figures and Tables

**Table 1:** Crystallographic data collection and refinement statistics.

Data Collection and Refinement Statistics (Molecular Replacement)	
CNP058-Bim BH3 domain	
**Data collection**	
Space group	C121
No. of molecules in asymmetric unit	1 + 1
Cell dimensions	
*a*, *b*, *c* (Å)	73.79, 34.67, 71.73
α, β, γ, (°)	90.00, 114.92, 90.00
Wavelength (Å)	0.9537
Resolution (Å)	33.46–2.45 (2.55–2.45)
No. unique reflections	6021 (703)
*R*_sym_ or *R*_merge_	0.097 (0.716)
*I*/σ*I*	6.1 (1.1)
CC_1/2_	0.99 (0.45)
Wilson B-factor	40.9
Completeness (%)	97.2 (98.4)
Redundancy	2.7 (2.7)
**Refinement**	
Resolution (Å)	2.45
No. reflections	6017
*R*_work_/*R*_free_	0.2126/0.2453
No. atoms	
Protein	1340
Water	10
*B*-factors	
Protein	52.91
Water	56.11
R.m.s. deviations	
Bond lengths (Å)	0.003
Bond angles (°)	0.56
Ramachandran statistics (%)	
Favored	97.42
Allowed	2.58
Disallowed	0.00

Values in parentheses are for highest-resolution shell.

**Table:** 

	Poxviral Bcl-2							
Pro-Death	SPPV14	M11L	MVA_F1	VAR_F1	DPV022	FPV039	CNP058	N1
**Bad**	>2000	>1000	NB	NB	NB	653	NB	>1000
**Bid**	341	100	NB	3200	NB	2	50	152
**Bik**	>2000	>1000	NB	NB	NB	30	NB	n/a
**Bim**	26	5	250	NB	340	10	353	72
**Bmf**	67	100	NB	NB	NB	16	294	n/a
**Hrk**	63	>1000	NB	NB	NB	24	312	n/a
**Noxa**	>2000	>1000	NB	NB	NB	28	3284	n/a
**Puma**	65	>1000	NB	NB	NB	24	2484	n/a
**Bak**	46	50	4300	2640	6930	76	508	71
**Bax**	32	75	1850	960	4040	76	326	n/a
**Beclin-1**	n/a	n/a	n/a	n/a	NB	n/a	n/a	n/a

**Table:** 

	Asfarviral Bcl-2		Herpesviral Bcl-2	
	A179L	BHRF1	Ks-Bcl-2	M11
**Bad**	258	>2000	>1000	NB
**Bid**	26	109	112	232
**Bik**	190	>2000	>1000	NB
**Bim**	6	18	29	131
**Bmf**	254	>2000	>1000	300
**Hrk**	1487	>1000	>1000	719
**Noxa**	1575	>2000	>1000	132
**Puma**	31	70	69	370
**Bak**	29	150	<50	76.3
**Bax**	26	1400	980	690
**Beclin-1**		n/a	n/a	40

**Table:** 

	Human Bcl-2					Sponge Bcl-2
	Bcl-2	Bcl-w	Bcl-x_L_	Mcl-1	A1	BHP2
**Bad**	16	30	5.3	>100,000	15,000	NB
**Bid**	6800	40	82	2100	1	NB
**Bik**	850	12	43	1700	58	NB
**Bim**	2.6	4.3	4.6	2.4	1	NB
**Bmf**	3	9.8	9.7	1100	180	NB
**Hrk**	320	49	3.7	370	46	3760
**Noxa**	>100,000	>100,000	>100,000	24	20	NB
**Puma**	3.3	5.1	6.3	5	1	NB
**Bak**	>1000	500	50	10	3	66
**Bax**	100	58	130	12	n/a	NB
**Beclin-1**	n/a	n/a	2300	n/a	n/a	n/a
